# Evaluation of the efficiency and safety of TransPRK and FS-LASIK refractive procedures on patients with astigmatism and amblyopia

**DOI:** 10.22336/rjo.2023.44

**Published:** 2023

**Authors:** George Sima, Cătălina-Ioana Tătaru, Mihnea Munteanu

**Affiliations:** * Department of Ophthalmology, “Victor Babeş” University of Medicine and Pharmacy Timișoara, Faculty of Medicine, Timişoara, Romania; ** Department of Ophthalmology, “Carol Davila” University of Medicine and Pharmacy, Bucharest, Romania; Alcor Ophthalmology Clinic, Bucharest, Romani

**Keywords:** FS-LASIK, TransPRK, astigmatism, refractive amblyopia, safety

## Abstract

**Purpose:** To compare the outcomes of transepithelial photorefractive keratectomy (transPRK) with femtosecond laser assisted in situ keratomileusis (FS-LASIK) for the correction of astigmatism on amblyopic eyes.

**Methods:** The design was a retrospective interventional study on 37 eyes with hyperopic or mixed astigmatism and refractive amblyopia, which underwent transPRK or FS-LASIK. The patients were distributed into 2 groups according to the technique used. Data was collected from patient files and comparison between groups was performed. The main outcomes measured were corrected distance visual acuity (CDVA), sphere, cylinder, spherical equivalent (SEQ), efficiency and safety indexes.

**Results:** In the transPRK group, SEQ improved significantly after 1 month, from 2.08 ± 2.02D (p<0.01) to 0.125 ± 0.86D and at the 12 month visit to -0.04 ± 0.62D (p>0.05), sphere improved from 4.03 ± 1.44D preoperatively to 0.67 ± 0.9D at 1 month (p<0.05) and further to 0.44 ± 0.71 at 12 months (p<0.05). CDVA improved from 0.194 ± 0.11 logMAR to 0.115 ± 0.1 logMAR at the 1-year visit. Safety index after 1 month was 1.09 ± 0.2 and 1.12 ± 0.35 at the 1-year visit. Efficiency index was 0.95 ± 0.22 at 1 month and 1.03 ± 0.34 after 1 year. In FS-LASIK group, SEQ improved after 1 month, from 2.28 ± 3.04 to -0.79 ± 0.73D (p<0.01), and further to -0.49 ± 0.79 (p>0.05) at the 12 month visit, sphere improved from 4.11 ± 2.35D preoperatively to -0.42 ± 0.66D at 1 month (p<0.05) and further to -0.08 ± 0.75D at 12 months (p<0.05). CDVA also improved from 0.191 ± 0.1 logMAR to 0.140 ± 0.1 logMAR at 1 year. Safety index after 1 month was 1.1 ± 0.2 and 1.16 ± 0.21 at the 1-year visit. Efficiency index was 0.98 ± 0.27 at 1 month and 1.06 ± 0.23 after 1 year.

**Conclusion:** Both procedures were safe and efficient in improving visual acuity for patients with mixed and hyperopic astigmatism and refractive amblyopia.

**Abbreviations: **transPRK = transepithelial photorefractive keratectomy, FS-LASIK = femtosecond laser in situ keratomileusis, logMAR = logarithm of the Minimum Angle of Resolution, BCVA = best corrected distance visual acuity, CDVA = corrected distance visual acuity

## Introduction

Refractive errors (myopia, hyperopia and astigmatism) are the most common eye diseases worldwide. If they are not corrected effectively and at the right age, they can cause social and economic problems for both patients and their families [**1**]. In 2019, WHO found that ametropia is responsible for severely impairing distance vision in 123.7 million people worldwide [**2**]. Notably, according to a study conducted in 2023 in the general population, the prevalence of astigmatism ranged from 6-62%, with higher rates in people over 70 years old [**3**]. Some degree of astigmatism is present in over 80% of the world’s population and is often considered physiological (<1D). When its magnitude exceeds the physiological threshold, uncorrected astigmatism can cause diplopia, glare, ghosting, blurred vision and astigmatism in adults. It can also cause meridional amblyopia in the paediatric population, so it is imperative to correct it as soon as possible to avoid this consequence. A deficient foveolar or peripheral retinal stimulation or inadequate binocular interaction has been suggested as a mechanism of occurrence [**4**]. This can also be caused by the presence of an anisometropia. Therapeutic strategies aim to achieve optimally corrected visual acuity in these eyes during the critical period when binocular vision is developing and to stimulate these eyes by occluding the congenital eye or by penalization [**5**]. The critical period corresponds to the period when stimulus deprivation occurs. The recovery period during which visual acuity may improve and which is not limited to the critical period is also of major importance. It can extend into puberty or even adulthood [**6**].

Studies have demonstrated an improved quality of vision in adult patients with refractive myopia who have undergone various refractive surgery techniques. In recent years, with the development of modern laser-assisted refractive surgery techniques such as laser in situ keratomileusis (LASIK) or photorefractive keratectomy (TransPRK), which have certain advantages over their classical variants, their potential to treat anisometropia in adults or to improve suboptimal astigmatic correction achieved by wearing contact lenses or spectacles has been suggested [**5**,**7**].

The aim of this study was to evaluate the visual parameters (CDVA, SE of refractive error, cylindrical diopter), efficiency and safety of implementing these techniques in amblyopic patients with hyperopic or mixed astigmatism and adult age.

## Methods

 The study design was retrospective, interventional and was conducted on a cohort of 37 eyes, of 28 patients diagnosed with refractive amblyopia. Patients were distributed according to the procedure used as follows: 14 eyes in the StreamLight group and 23 eyes in the FS-LASIK group.

Inclusion criteria: stable diopters in the last year, age over 18 years, hyperopic or mixed astigmatism, amblyopia, willingness to undergo refractive surgery, CDVA ≤ 0.8 decimal, either sex.

Exclusion criteria: pre-existing ocular pathologies (glaucoma, uveitis, optic media opacities, retinopathies), pre-existing dry eye syndrome, keratoconus, keratoconus fruste forms, collagen diseases, corneal stromal dystrophies, Fuchs corneal dystrophy, history of chronic medication that might affect the cornea, autoimmune diseases, uncontrolled diabetes mellitus, pregnancy or lactation, single functional eye.

Preoperative evaluation included BCVA using ETDRS optotype and converted to logMAR for further statistical analysis, refractometry + cycloplegia, non-contact tonometry, anterior pole slit lamp examination, fundus examination, topography + pupillary evaluation (Oculyzer + Topolyzer Vario II), and Schirmer test. The efficiency index was defined as the ratio of postoperative UCVA/preoperative BCVA and the safety index was defined as the ratio of postoperative BCVA/preoperative BCVA.

The procedures were performed using the Wavelight FS 200 femtosecond laser platforms as well as the EX500 excimer laser from 2018-2021 and targeted emmetropia as refractive outcome. All procedures were performed by the same surgeon, and the patients expressed their consent to undergo the surgery and have their personal information processed for scientific purposes. The study was conducted in accordance with the principles of the Declaration of Helsinki and was approved by the Ethics Committee of Alcor Ophthalmology Clinic. At the end of the surgery, patients operated on by transPRK were fitted with a therapeutic contact lens, which was removed after 7 days. The postoperative regimen for all patients consisted of antibiotics + steroidal anti-inflammatories (Tobramycin + Dexamethasone) 6 times/day for the first 10 days, with progressive dose reduction up to 1 month, preservative-free ocular lubricant eye drops for 1 month (first 3 days every hour) and non-steroidal anti-inflammatories (Ibuprofen 200mg) p. o. 3 times/day for the first 3 days, and StreamLight patients were also offered painkillers p.o. 2 times/day for the first 2 postoperative days.

Statistical analysis of the data was performed using SPSS software (ver. 26.0, SPSS Inc., Chicago, Illinois, USA). Normality of data was tested using the Shapiro-Wilk test, comparison of means of pre- and postoperative values using the paired samples t-test and Mann-Whitney U test, respectively, and correlations were tested using Pearson/Spearman correlation. The level of statistical significance considered in all cases was p<0.05.

## Results

The mean age of the patients in the Streamlight group was 24.07 ± 5.04 years (range 19 to 40 years) and the gender distribution was 57.14% females and 42.86% males. Of the 14 cases included, 4 had hyperopic astigmatism, 8 had mixed astigmatism.

The mean age of the patients in the FS-LASIK group was 26.39 ± 5.50 years (from 21 to 44 years), and the gender distribution was 52.17% females, 47.83% males, and the types of astigmatism were 12 compound hyperopic, 11 mixed.

Preoperative visual parameters are presented in **Table 1**.

**Table 1:** Preoperative parameters for both groups

*Parameter*	TransPRK	FS-LASIK	*p-value*
*Spherical Equivalent (D*	2.08 ± 2.02 (-0.625 to 4.875)	2.28 ± 3.04 (-2.75 to 7.625)	0.813
*Sphere (D)*	4.03 ± 1.44 (1.25 to 6.5)	4.11 ± 2.35 (0.5 to 8.25)	0.894
*Cylinder (D)*	-3.89 ± 2.35 (-6.50 to -1)	-3.64 ± 1.83 (-7 to -1)	0.698
*CDVA (logMAR)*	0.194 ± 0.11	0.191 ± 0.109	0.975

According to Shappiro-Wilk test, age and CDVA were not normally distributed, p<0.05. The rest of the parameters were normally distributed, p>0.05. Independent samples T-test showed that there were no significant differences between the means of the evaluated parameters. p>0.05 in all cases. Independent samples T-test showed no significant differences between the efficiency or safety of the two procedures at either 1 month or 12 months.

TransPRK: A statistically significant reduction was observed in the spherical equivalent, from 2.08D to 0.125D, in the spherical diopter, from 4.03D to 0.67D, and in the cylindrical diopter, from 3.89D to -1.1D (p<0.05), with a tendency to emmetropia, at one month postoperatively. At the 6-month follow-up, we found an additional improvement in CDVA, to 0.161log MAR, but statistically insignificant. At the end of the follow-up period, all the parameters evaluated changed slightly since the previous visit, but only the spherical and cylindrical diopter were statistically significant (p<0.05) (**Table 2**).

**Table 2:** Changes in parameters assessed over the entire monitoring period - transPRK

			TransPRK				
*Parameter*	Preop	1 month	*p-value (preop-1m)*	6 months	*p-value (1m-6m)*	12 months	*p-value (6m-1y)*
*SEQ (D)*	2.08 ± 2.02	0.125 ± 0.86	0.003*	0.142 ± 0.71	0.893	-0.04 ± 0.62	0.153
*Sphere (D)*	4.03 ± 1.44	0.67 ± 0.9	<0.001*	0.69 ± 0.91	0.885	0.44 ± 0.71	0.047*
*Cylinder (D)*	-3.89 ± 1.92	-1.1 ± 0.63	<0.001*	-1.1 ± 0.59	1	-0.87 ± 0.43	0.017*
*BCVA/CDVA (logMAR)*	0.194 ± 0.11	0.161 ± 0.1	0.178	0.143 ± 0.11	0.381	0.115 ± 0.1	0.189
* statistical significance; m = month; y = year							

FS-LASIK: At one-month postoperatively, control spherical equivalent, cylindrical diopter, as well as spherical diopter changed statistically significant compared to preoperative values (p<0.01), with a tendency to emmetropia. Subsequently, at the 6-month control, both spherical and cylindrical diopters changed significantly towards emmetropia (p<0.01) and UCVA (p<0.05). By the 12-month visit, the trend towards emmetropia remained statistically insignificant.

**Table 3:** Changes in parameters assessed over the entire monitoring period - FS-LASIK

			FS-LASIK				
*Parameter*	Preop	1 month	*p-value (preop-1m)*	6 months	*p-value (1m-6m)*	12 months	*p-value (6m-1y)*
*SEQ (D)*	2.28 ± 3.04	-0.79 ± 0.73	<0.001*	-0.59 ± 0.63	0.009*	-0.49 ± 0.79	0.277
*Sphere (D)*	4.11 ± 2.35	-0.42 ± 0.66	<0.001*	-0.18 ± 0.77	0.007*	-0.08 ± 0.75	0.274
*Cylinder (D)*	-3.64 ± 1.83	-0.78 ± 0.51	<0.001*	-0.81 ± 0.4	0.741	-0.81 ± 0.44	1
*BCVA/CDVA (logMAR)*	0.191 ± 0.1	0.186 ± 0.16	0.869	0.146 ± 0.1	0.014*	0.140 ± 0.1	0.46
* statistical significance; m = month; y = year							

**Fig. 1:**
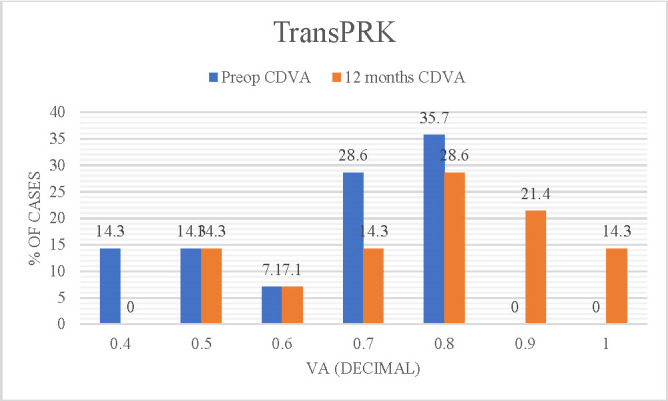
Comparative graph of preoperative optimally corrected (decimal) visual acuity (BCVA) and postoperative corrected visual acuity (CDVA) - transPRK technique

**Fig. 2:**
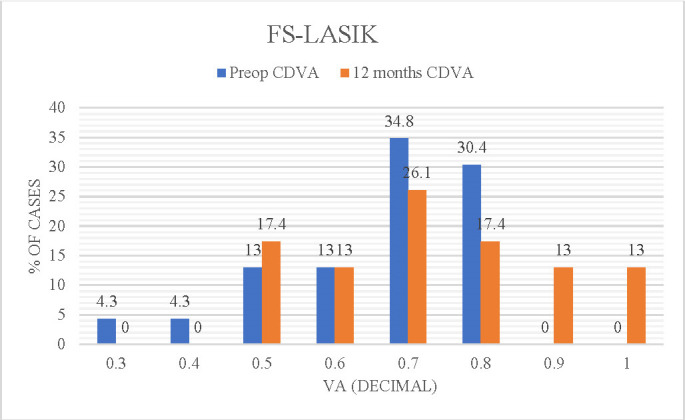
Comparative graph of preoperative optimally corrected (decimal) visual acuity (BCVA) and postoperative corrected visual acuity (CDVA) - FS-LASIK technique

**Fig. 1** and **2** present the improvement in visual acuity (CDVA) at 12 months post-intervention compared to preoperative visual acuity values (CDVA).

**Fig. 3:**
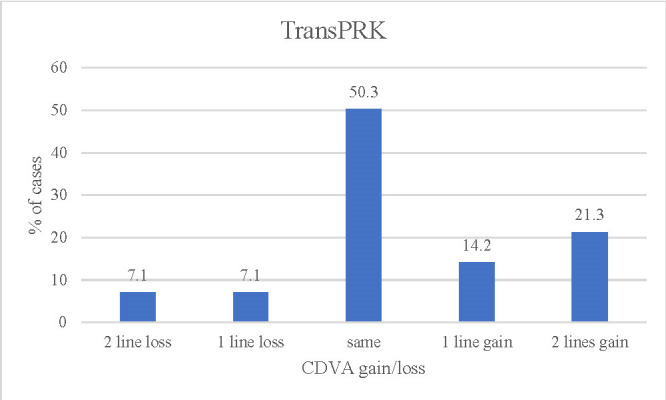
CDVA lines (decimal) gain/loss compared to preoperative CDVA for transPRK

**Fig. 4:**
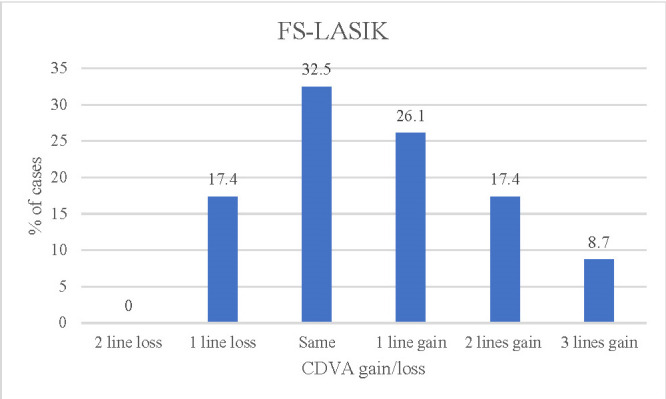
CDVA lines (decimal) gain/loss compared to preoperative CDVA for FS-LASIK

**Fig. 3** and **4** show the changes in UDVA in each group at 12 months. In the transPRK group, 21.3% gained 2 lines of UDVA compared to preoperative CDVA values. The cases operated on by FS-LASIK gained 26.1% - 1 line of visual acuity (decimal) and the same proportion gained 2 or more lines.

Both procedures proved safe at the one-month postoperative follow-up, in which the safety index in both groups exceeded the threshold value of 0.85, with no statistically significant differences between them (p>0.05). Neither eye lost >2 BCVA lines. In terms of efficiency, both procedures showed similar efficiency indices at both visits (1 month and 12 months), with no statistically significant differences (**Table 4**).

**Table 4:** Comparison of efficiency and safety indices at 1 month and 12 months between the two techniques

Efficacy	StreamLight	FS-LASIK	*p-value*
*1 month*	0.95 ± 0.22	0.98 ± 0.27	0.584
*12 months*	1.03 ± 0.34	1.06 ± 0.23	0.392
Safety index			
*1 month*	1.09 ± 0.2	1.1 ± 0.2	0.852
*12 months*	1.12 ± 0.35	1.16 ± 0.21	0.305

**Fig. 5:**
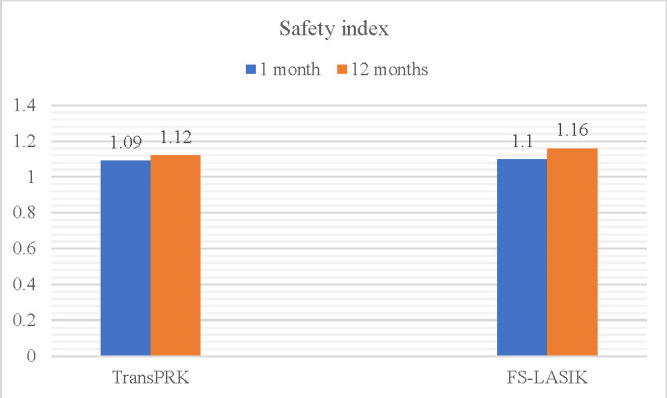
Comparative evolution of safety indexes between procedures

**Fig. 6:**
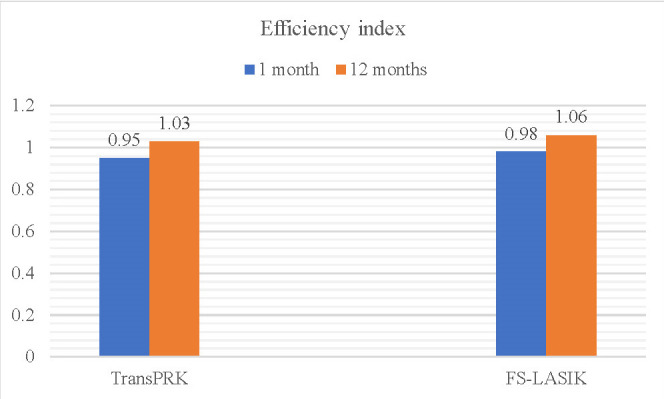
Comparative evolution of efficiency indexes between procedures

During the follow-up period, both the safety and efficiency indices increased discretely, but also without significant differences between groups (**Fig. 5**,**6**).

## Discussions

Although there are numerous studies that have examined amblyopia correction by refractive surgery in the pediatric population [**8**,**9**], those that target the adult population are fewer [**10**,**11**]. In the current study, we evaluated the postoperative refractive outcomes, efficiency and safety of performing refractive surgery in the adult population with refractive amblyopia. Both mixed and hyperopic astigmatism are difficult to correct completely using aerial correction and even more difficult with contact lenses.

Recovery is slower than for myopic astigmatism, which is why we considered evaluating the first follow-up visit in our study at 1 month (not the immediate postoperative visit) more important, during which time the corneal epithelium has regenerated and reorganized (in transPRK patients), common transient symptoms have diminished, and the postoperative treatment regimen has been reduced.

In our limited retrospective study, we found a significant improvement in UDVA and CDVA postoperatively in both techniques, visual acuity close to emmetropia, as well as good safety and efficiency indices in accordance with international publications [**12**]. In evolution, they were relatively stable, improving further, slowly, up to 12 months. Consistent with studies published in literature, FS-LASIK has been shown to be effective and safe in the treatment of astigmatism with discretely higher indices, but without statistical significance compared to transPRK. There was no regression of astigmatic correction at 12 months in either group, possibly due to a slight intentional overcorrection in the immediate postoperative period. We found that overall, in patients undergoing FS-LASIK, 26.1% had a gain of two or more uncorrected VA (visual acuity) lines, and those undergoing transPRK had a 21% gain of two VA lines at 12 months. This final improvement in visual acuity, gained by the patients operated on in the current study, suggests that in some cases, astigmatic amblyopia may be more of an optic problem than truly amblyopic, but certainly the mechanism of these gains remains incompletely elucidated. Corneal toricity is significantly improved following laser refractive procedures, so improving the quality of the entire ocular diopter may play a role. Alper Agca et al. [**13**] identified similar improvements, with 26% of all amblyopic patients treated with LASIK gaining 2 VA lines, but the cohort including myopic astigmatism. There are various reports on the improvement of CDVA. Cagil et al. [**14**] showed a gain of 3 or more lines in 25% of the cases treated. Using PRK, Roszkowska et al. [**15**] suggested gains of 1 line of VA in 50% of patients, 21% gains of 2 lines, and 12% gains of 3 lines. In contrast, Sakatani et al. [**16**] did not report VA improvements in the astigmatic eye group, but only in myopic eyes, however on a small number of patients (3 patients with mixed astigmatism).

The healing process in FS-LASIK is faster than in TransPRK [**17**], thus explaining the significant differences in visual acuity gain, spherical and cylindrical diopter equivalents in the 1 month to 6 months interval. Because healing and visual recovery post LASIK or transPRK occurs in the first postoperative months, improvement by 12 months may suggest the role of neurosensory adaptive mechanisms, although the plasticity of the visual system in adulthood is thought to act as a barrier to this. Despite this plasticity, some studies suggest a benefit of treating amblyopia in adulthood [**11**,**18**]. Amblyopia can be treated in younger patients who could theoretically still be in recovery, and, in older patients, the mechanism was probably the improvement of the otherwise suboptimal correction achieved by glasses/contact lens correction. 

The limitations of the study were its retrospective nature, the small cohort analyzed, which is why it was impossible to subdivide the astigmatism according to the size of the defect, which would have provided additional information on the efficiency of the procedures. 

## Conclusions

In conclusion, both techniques can positively influence the visual prognosis of patients with refractive amblyopia, having good safety profiles and efficiency.

**Conflict of Interest statement**

None.

**Informed Consent and Human and Animal Rights statement**

Informed consent was obtained from all participants included in this study.

**Authorization of the use of human subjects**

The study was performed in compliance to Helsinki Declaration and has been approved by the Ethics Committee of Alcor Ophthalmology Clinic, Bucharest, Romania.

**Acknowledgements**

None.

**Sources of Funding**

None.

**Disclosures**

None.
